# The relationship between pregnancy stress and mental health of the pregnant women: the bidirectional chain mediation roles of mindfulness and peace of mind

**DOI:** 10.3389/fpsyg.2023.1295242

**Published:** 2024-01-08

**Authors:** Shasha Sun, Chunqi Luo, Xun Zeng, Qichang Wu

**Affiliations:** ^1^College of Humanities and Social Sciences, Zhongkai University of Agriculture and Engineering, Guangzhou, China; ^2^The First Affiliated Hospital of Sun Yat-sen University, Guangzhou, China

**Keywords:** pregnant women, mental health, pregnancy stress, mindfulness, peace of mind, positive psychological quality

## Abstract

**Objective:**

This study aimed to investigate the relationship between pregnancy stress and mental health of the pregnant women, employing a positive psychology perspective. Specifically, the study sought to explore how the two positive psychological qualities of mindfulness and peace of mind may serve as potential mediators in the association between pregnancy stress and mental health of the pregnant women.

**Methods:**

Seven hundreds and thirteen pregnant women seeking care at the First Affiliated Hospital of Sun Yat-Sen University were included in this study. The participants completed a self-report demographic questionnaire, as well as several validated scales including the Pregnancy Pressure Scale (PPS), Mindful Attention Awareness Scale (MAAS), Peace of Mind Scale (PoMS), and Chinese Health Questionnaire (CHQ). The Amos 23.0 system was utilized to construct structural equation models.

**Results:**

A total of 713 participants had an average age of 29.46 ± 4.81 years and an average gestational age of 24.26 ± 22.66 weeks. Out of the pregnant women, 163 (22.9%) experienced moderate or higher levels of pregnancy stress (PPS > 1), while 212 (29.7%) exhibited mental distress (CHQ > 3). Pregnancy stress exhibited a positive association with mental distress, while displaying negative associations with mindfulness and peace of mind. Mindfulness and peace of mind were negatively associated with mental distress. By employing structural equation modeling, the analysis revealed that mindfulness and peace of mind acted as partial mediators in the relationship between pregnancy stress and mental health. Furthermore, the identified models exhibited bidirectional sequential mediating pathways, suggesting that the pathways of mindfulness ↔ peace of mind mitigated the harmful influence of pregnancy stress on the mental health of pregnant women.

**Conclusion:**

This study adds to the current body of knowledge by investigating the relationships among mindfulness, peace of mind, pregnancy stress, and mental health in pregnant women. From a positive psychology framework, it provides valuable understanding of the intricate dynamics between pregnancy stress and protective factors of mental health. Consequently, interventions aimed at bolstering positive psychological qualities in pregnant women should prioritize the cultivation of mindfulness to foster peace of mind, or alternatively, the cultivation of peace of mind to enhance mindfulness, ultimately leading to improved mental health outcomes.

## Introduction

The period of pregnancy is marked by joy and significance as women prepare for the arrival of their baby. Nonetheless, it can also induce stress due to the various physical, psychological, and interpersonal changes and burdens experienced by women (Furber et al., 2009; Brunton et al., 2022). Extensive research has demonstrated that stress during pregnancy can have detrimental effects on women’s mental health (Coussons-Read, 2013; Parftt and Ayers, 2014; Puertas-Gonzalez et al., 2022), thereby posing a global challenge in the perinatal period (Howard et al., 2014; Woody et al., 2017; Romero-Gonzalez et al., 2020). Notably, studies have revealed a high prevalence of mental distress among Chinese pregnant women [ranging from 7.9 to 68.4%, as reported by Hou et al. (2018) and Tang et al. (2019)]. The hormonal changes experienced by women during pregnancy have the potential to exacerbate mental distress and emotional instability. This period is crucial for providing care and support to their psychological well-being (Duncan et al., 2017; Tuxunjiang et al., 2023).

In order to mitigate prenatal stress and enhance the psychological well-being of pregnant women, positive psychology approaches have been suggested (Corno et al., 2018; Zhao et al., 2019; Shaghaghi et al., 2020; Rastad et al., 2021; Abbasi et al., 2022; Tuxunjiang et al., 2022, 2023). Among these approaches, mindfulness, which entails maintaining a non-judgmental awareness and acceptance of emotions, thoughts, and physical sensations in the current time, is considered a fundamental positive psychological quality (Bishop et al., 2004; Wallace and Shapiro, 2006; Kabat-Zinn, 2015). Research has demonstrated the efficacy of mindfulness interventions in mitigating stress and negative affect in pregnant women (Vieten and Astin, 2008; Dunn et al., 2012; Zhang et al., 2019). Additionally, the cultivation of mindfulness skills during pregnancy has been linked to a decrease in emotional distress experienced prenatally (Braeken et al., 2017; De Waal et al., 2023). Consequently, mindfulness may serve as a safeguard for mental health outcomes throughout the perinatal period.

Peace of mind (PoM) and closely related constructs, such as equanimity and serenity, are also important but underappreciated constructs in positive psychology (Cohrs et al., 2013; Floody, 2014; Yu et al., 2019; Juneau et al., 2020; Soysa et al., 2021; Niemiec, 2022; Wissing, 2022). Regrettably, these constructs are often overlooked in prevailing Western models of well-being (Delle Fave et al., 2022). PoM encompasses a dynamic psychological state characterized by inner tranquility and balance. It is achieved through self-regulation and spiritual development, leading to optimal cognitive functioning (Lee et al., 2013). PoM is considered an ideal state of low arousal and positivity in Chinese and other eastern cultures (Ward, 2010; Lee et al., 2013; Li and Cui, 2022). Within these cultural contexts, PoM is seen as a fundamental requirement for positive human experiences and overall well-being (Diener and Tov, 2012; Cohrs et al., 2013; Floody, 2014; Niemiec, 2022). Researches have shown that PoM and inner peace have significant impacts in reducing negative emotions (Lee et al., 2013; García-Jimenez et al., 2014; Ge et al., 2020; Liang et al., 2020; Soysa et al., 2021; Xi et al., 2021). Consequently, psychologists often consider inner peace and peace of mind as vital indicators of mental health. Positive emotions, as per the broaden-and-build theory, possess the capacity to expand cognitive processes, enhance cognitive adaptability, and facilitate effective resolution of problems (Fredrickson and Branigan, 2005). Based on the theory of conservation of resource (COR), people are motivated by strategic reasons to generate, amass, and safeguard valuable asserts (Halbesleben et al., 2014). Within Chinese culture, the expression of intense emotions is commonly perceived as a potential depletion of resources (Friedman et al., 2006). Individuals who possess elevate levels of PoM may experience a heightened sense of emotional stability and regulation, thereby avoiding excessive excitement or distress (Yu et al., 2019). Having access to a greater array of resources for stress management may enhance an individual’s capacity to effectively address stress and recover from challenges, thereby promoting psychological well-being. Consequently, it is hypothesized that the cultivation of a more adaptable, stable, and resource-conserving coping strategy through keeping peace of mind could potentially alleviate stress and enhance mental health among pregnant women. This would enable them to maintain emotional stability within a moderate range when confronted with stress during the unique period of pregnancy. To the best of our knowledge, no study has yet investigated the influence of PoM on the association between stress and mental health outcomes in this population.

Previous research has confirmed a strong correlation between mindfulness and PoM, indicating that an increase in mindfulness may result in an elevation of PoM (Liu et al., 2015; Xu et al., 2015; Dambrun et al., 2019; Liao et al., 2023; Xie, 2023). Both ancient Buddhism and contemporary meditation prioritize the attainment of inner peace through meditation, considering it as the initial step toward developing PoM (Ren et al., 2012; Desbordes et al., 2015; Liu et al., 2015; Weber, 2017). Practicing mindfulness encourages embracing one’s current experiences without any criticism or evaluation. The achievement of peace of mind becomes more feasible by embracing a non-critical mindset toward oneself and abstaining from assigning fault to one’s feelings, thoughts and sensations (Liu et al., 2015; Xu et al., 2015). Maintaining emotional equilibrium and inner peace is crucial for pregnant individuals who experience a mixture of anticipation and stress during their pregnancy, as it directly impacts their mental well-being. It is reasonable to hypothesize that practicing mindfulness and achieving a state of peace of mind would serve as sequential mediators in the association between pregnancy stress and mental health of the pregnant women.

Another potential hypothesis that has been suggested is that the pathway connecting PoM to mindfulness could also play a role in the relationship between pregnancy stress and mental health of the pregnant women. Mindfulness, which is characterized as purposeful and non-judgmental awareness (Kabat-Zinn, 2005), appears to require a preparatory phase for individuals to cultivate this state of mind. In contrast, PoM facilitates a more receptive and objective perception of experiences by adopting an attitude that neither resists nor clings to emotions or situations (Desbordes et al., 2015). This enables individuals to effectively navigate challenges, including stress during pregnancy, with a sense of calm and tranquility. Therefore, it can be inferred that PoM serves as a preparatory stage for the cultivation of mindfulness and serves as a significant mechanism for comprehending the beneficial impacts of mindfulness on overall well-being (Ekman et al., 2005; Juneau et al., 2019; Soysa et al., 2021). In other words, mindfulness develops as a function of PoM (Juneau et al., 2020; Rogers et al., 2021). The findings of Soysa et al. (2021) provide evidence for this notion, and they discovered that PoM not only predicted reduced stress levels but also enhanced mental well-being among undergraduate students, even when accounting for the influence of mindfulness. Consequently, it is plausible to suggest that PoM and mindfulness may act as sequential mediators in the relationship between stress and the mental health of pregnant women.

**Figure 1:**
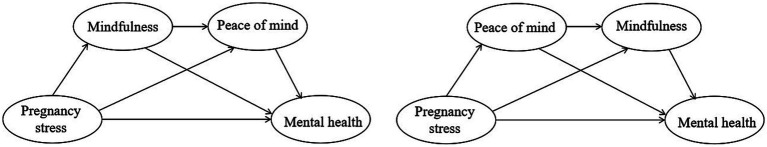
The hypothesized research model. **(A)** The hypothesized chain mediating effect of mindfulness and peace of mind in the relationship between pregnancy stress and the mental health of pregnant women. **(B)** The hypothesized chain mediating effect of peace of mind and mindfulness in the relationship between pregnancy stress and the mental health of pregnant women.

Based on what we currently know, there is a shortage of empirical evidence that comprehensively elucidates the collective impact of mindfulness and peace of mind on stress experienced during pregnancy and the psychological welfare of pregnant women. This study aims to examine the associations between pregnancy stress, mindfulness, peace of mind, and mental health of the pregnant women and potential mediating effect of mindfulness and peace of mind by investigating a sample of 713 individuals. The following research hypothesis is formulated: in the relationship between pregnancy stress and the mental health of pregnant women, mindfulness has a significant mediating effect; and peace of mind has a significant mediating effect; furthermore, mindfulness and peace of mind demonstrate a bidirectional chain effect. The hypothesized research models are shown in Figures 1A,B.

## Methods

### Participants and procedure

From January 2023 to July 2023, a convenient sampling method was employed to gather data from pregnant women seeking care at the First Affiliated Hospital of Sun Yat-Sen University in Guangzhou, Guangdong Province. Data collection took place during their waiting period. The inclusion criteria included: a definitive pregnancy diagnosis, age above 20, proficient reading and communication skills to independently complete the questionnaires, willingness to participate in the study, and provision of informed consent. Conversely, the exclusion criteria included: severe mental or physical disorders hindered questionnaire completion, severe pregnancy-related complications (such as gestational diabetes, heart disease and hypertension), high-risk pregnancies (fetal malformation, pelvic abnormalities, etc.), and indications for cesarean section. After providing their informed consent, the participants were directed to scan the QR code generated by the Wenjuanxing platform^1^ in order to complete the questionnaires using their personal smartphones. A total of 742 pregnant women completed the questionnaires, and 29 surveys were excluded due to insufficient response time or consistent patterns of response. Ultimately, 713 valid questionnaires were obtained, resulting in an effective rate of 96.09%.

## Measures

### Pregnancy pressure scale

The PPS (Chen et al., 1989) is a comprehensive measure consisting of 30 items that assess four dimensions: parent role (e.g., Worry about whether I would be forced to give up work when I have a child.), mother and child health and safety (e.g., Worry about whether I will have a premature delivery.), body shape and physical activity change (e.g., Worry about getting too fat.), and other factors (e.g., Worry about not being able to provide good living conditions for the child.). Each item is scored on a 4-point Likert scale ranging from 0 (no pressure) to 3 (severe pressure). The total score of the scale is calculated by summing the scores of all items and dividing it by the total number of items (Chen et al., 2021). Scores falling within the ranges of 0, 0.001–1, 1.001–2, and 2.001–3 indicate no, mild, moderate, and severe pressure, respectively (Kim and Chung, 2018). In this study, the average PPS score of the 713 participants was 0.67 ± 0.54, and the four dimension scores were 0.38 ± 0.42, 0.91 ± 0.68, 0.77 ± 0.76 and 0.63 ± 0.65, respectively. Additionally, the Cronbach’s alpha coefficient for the MAAS in this study was found to be 0.953.

### Mindful attention awareness scale

The MAAS (Brown and Ryan, 2003) is a 15-item instrument designed to assess trait mindfulness (e.g., I find it difficult to stay focused on what’s happening in the present.). Participants rate each item on a 6-point Likert scale, which spams from 1 (almost always) to 6 (almost never). Greater levels of trait mindfulness are indicated by higher scores on the MAAS. In this study, the average MAAS score among the 713 participants was 4.40 ± 0.69. Additionally, the Cronbach’s alpha coefficient for the MAAS in this study was found to be 0.868.

### Peace of mind scale

The PoMS (Lee et al., 2013) is a 7-item measure used to evaluate the extent of peace of mind experienced by participants in everyday lives (e.g., “I have peace and harmony in my mind.” “It is difficult for me to feel settled.”). Participants evaluate each item using a 5-point Likert scale, ranging from 1 (not at all) to 5 (all of the time). Higher scores on the PoMS indicate higher levels of peace of mind. In this study, the average PoMS score among the 713 participants was 3.75 ± 0.61. Additionally, the Cronbach’s alpha coefficient for the MAAS in this study was found to be 0.976.

### Chinese health questionnaire

The CHQ (Cheng and Williams, 1986) is a validated clinical instrument consisting of 12 items designed to assess mental health and distress among Chinese adults (thought to have taken into account the Chinese ways of expressing emotional distress), primarily for the screening of minor psychiatric morbidity in the community and primary care settings (e.g., “been suffering from headache or pressure in your head?” “been getting along well with your family and close relatives?” “been feeling that life is entirely hopeless?”). Participants rate each item on a 4-point scale with a 0 (not at all) - 0 (the same as usual)- 1 (more than usual) - 1 (a lot more than usual) format. Higher scores on the CHQ indicate greater levels of mental distress in pregnant women. In this study, the average CHQ score among the 713 participants was 3.20 ± 0.43. Additionally, the Cronbach’s alpha coefficient for the CHQ in this study was found to be 0.811.^2^

### Data analysis

The statistical analyzes conducted in this study included descriptive statistics, independent sample *t*-test, analysis of ANOVA, correlational analysis, and computation of Cronbach’s α using SPSS 27.0. The data’s normality was evaluated by conducting the Shapiro–Wilk test, with a significance level of *p* > 0.05. The Amos 23.0 modeling and analysis system was utilized to examine the mediating effect and generate an analysis diagram illustrating the relationship between the variables. The association between pregnancy stress and mental health of the pregnant women was investigated using Structural Equation Modeling (SEM), which also explored the mediating role of mindfulness and peace of mind. Additionally, the research examined the consecutive intermediary impacts of mindfulness → peace of mind, and peace of mind → mindfulness, in the correlation between stress during pregnancy and the mental well-being of the expectant mothers. The importance of these intermediary impacts was evaluated through a non-parametric percentile Bootstrap procedure, with a bootstrap sample size of 5,000. The ultimate analysis model was derived by iteratively adjusting the level of model fitting. The model’s goodness of fit was assessed using the following criteria: CMIN/DF should be less than 5, standardized root-mean-square residual (SRMR) should be less than 0.08, root-mean-square error of approximation (RMSEA) should be less than 0.08, goodness of fit index (GFI) should be greater than 0.90, and comparative fit index (CFI) should be greater than 0.95.

## Results

### Common method bias test

According to Harman’s one-factor test, the initial factor accounted for 25.13% of the overall variance, which was lower than the predetermined threshold of 40%. According to Podsakoff et al. (2003), it can be inferred that the findings of this study were not significantly affected by the presence of common method bias.

### Demographic data

A total of 713 participants had an average age of 29.46 ± 4.81 years and an average gestational age of 24.26 ± 22.66 weeks. As shown in Table 1, 681 (95.51%) identified as Han nationality, while 32 (4.49%) belonged to ethnic minorities. A total of 149 women (20.90%) were below the age of 25, while 480 women (67.32%) fell within the age range of 25 to 35. Additionally, 84 women (11.78%) were older than 35. Regarding educational attainment, 122 women (17.11%) had not completed high school, 138 women (19.35%) had a high school education, 398 women (55.82%) held a bachelor’s degree, and 55 women (7.71%) possessed a master’s degree or higher. The employment status of the participants revealed that 562 women (78.82%) were employed, while 151 women (21.18%) identified as housewives. Furthermore, 185 women (25.95%) resided in rural areas, while 528 women (74.05%) lived in urban areas. Lastly, the marital status of the participants revealed that 695 women (97.48%) were married while 18 (2.52%) unmarried. A total of 421 women (59.05%) were experiencing their initial pregnancy, whereas 292 women (40.95%) had previously given birth. Out of the entire sample, 450 women (63.11%) had intentionally conceived, while 263 women (36.89%) had unplanned pregnancies. Among the 263 women, only a mere 7 women (0.98%) expressed feelings of burden associated with their unplanned pregnancies, while the remaining 256 women (35.91) wholeheartedly embraced their offspring.

**Table 1:** Demographic characteristics, PPS, CHQ, MAAS, and PoMS scores of pregnant women with varying demographic attributed.

Variable	Number (*n*)	Percent (%)	PPS	MAAS	PoMS	CHQ
Nationality						
Ethnic Han	681	95.51	0.68 ± 0.53	4.40 ± 0.69	3.74 ± 0.61	2.95 ± 2.06
Minority	32	4.49	0.54 ± 0.62	4.48 ± 0.81	3.88 ± 0.68	2.50 ± 2.50
*t*	--	--	1.443	−0.617	−1.220	1.193
*p*	--	--	0.149	0.537	0.223	0.233
Age						
≤25	149	20.90	0.81 ± 0.61a	4.33 ± 0.66	3.63 ± 0.63b	3.06 ± 2.04
26 ~ 35	480	67.32	0.66 ± 0.54b	4.42 ± 0.72	3.78 ± 0.61a	2.88 ± 2.14
>35	84	11.78	0.56 ± 0.39b	4.47 ± 0.63	3.72 ± 0.55	2.93 ± 2.08
*F*	--	--	6.902	1.100	3.338	0.393
*p*	--	--	0.001	0.333	0.036	0.675
Education						
Lower than high school	122	17.11	0.57 ± 0.47b	4.27 ± 0.64	3.54 ± 0.63c	3.19 ± 2.27
High school or technical secondary	138	19.35	0.74 ± 0.60a	4.40 ± 0.72	3.62 ± 0.58b	2.74 ± 1.84
Bachelor	398	55.82	0.68 ± 0.54	4.43 ± 0.71	3.83 ± 0.62a	2.93 ± 2.12
Master and above	55	7.71	0.70 ± 0.57	4.47 ± 0.60	3.87 ± 0.45a	2.80 ± 1.86
*F*	--	--	2.195	1.857	9.871	1.089
*p*	--	--	0.087	0.136	0.000	0.353
Monthly family income						
≤10,000	74	10.39	0.89 ± 0.71a	4.14 ± 0.79c	3.46 ± 0.72c	3.92 ± 2.52a
10,001 ~ 20,000	161	22.58	0.70 ± 0.51b	4.36 ± 0.72b	3.70 ± 0.58b	2.90 ± 1.93b
20,001 ~ 30,000	213	29.87	0.70 ± 0.54b	4.39 ± 0.65b	3.70 ± 0.64b	2.97 ± 2.17b
>30,000	265	37.17	0.61 ± 0.49b	4.50 ± 0.67a	3.89 ± 0.54a	2.64 ± 2.08c
*F*	--	--	5.340	5.502	11.905	7.564
*p*	--	--	0.001	0.000	0.000	0.000
Occupation						
Full-time job	562	78.82	0.68 ± 0.55	4.44 ± 0.70	3.77 ± 0.63	2.92 ± 2.07
Housewives	151	21.18	0.64 ± 0.50	4.25 ± 0.67	3.64 ± 0.56	2.93 ± 2.10
*t*	--	--	0.804	2.991	2.417	0.009
*p*	--	--	0.421	0.003	0.016	0.993
Living site						
Village	185	25.95	0.73 ± 0.56	4.34 ± 0.72	3.68 ± 0.64	2.82 ± 2.00
City	528	74.05	0.66 ± 0.53	4.42 ± 0.69	3.77 ± 0.60	3.24 ± 2.29
*t*	--	--	1.658	−1.262	−1.740	−2.360
*p*	--	--	0.098	0.207	0.082	0.019
Marital status						
Married	695	97.48	0.67 ± 0.54	4.40 ± 0.70	3.76 ± 0.61	2.91 ± 2.05
Unmarried or other (except for divorced)	18	2.52	0.85 ± 0.74	4.26 ± 0.69	3.32 ± 0.78	3.67 ± 2.89
*t*	--	--	−1.385	0.784	3.101	−1.527
*p*	--	--	0.166	0.383	0.003	0.127
First birth						
Yes	421	59.05	0.76 ± 0.58	4.41 ± 0.70	3.80 ± 0.63	2.97 ± 2.07
No	292	40.95	0.55 ± 0.46	4.38 ± 0.68	3.66 ± 0.59	2.87 ± 2.09
*t*	--	--	5.095	0.053	2.953	0.663
*p*	--	--	0.000	0.593	0.003	0.507
Planned pregnancy						
Yes	450	63.11	0.64 ± 0.51c	4.45 ± 0.69a	3.82 ± 0.59a	2.85 ± 2.00
No, but embrace it	256	35.91	0.73 ± 0.59b	4.34 ± 0.69b	3.64 ± 0.62b	3.03 ± 2.17
No, feel burdened	7	0.98	1.01 ± 0.71a	3.64 ± 0.71c	3.00 ± 1.06c	4.14 ± 3.39
*F*	--	--	3.767	6.438	12.005	1.823
*p*	--	--	0.024	0.002	0.000	0.162

The letters a, b, and c following the Mean ± SDs indicate statistically significant differences among the various levels of the variables. ps < 0.05. (*N* = 713).

### Pregnancy stress, mindfulness, peace of mind and mental health level across various demographic characteristics

Among the 713 individuals who took part in study, 163 individuals (22.9%) exhibited moderate or higher levels of pregnancy stress (PPS > 1), while 212 participants (29.7%) experienced mental distress (CHQ > 3). Statistically significant variations in PPS scores were observed among pregnant females of varying age groups, educational backgrounds, monthly family income brackets, as well as parity and pregnancy intention. Significant variations in MAAS scores were found among pregnant females of varying monthly family incomes, occupational statuses and pregnancy intention. Significant variations in PoMS score were found among pregnant females with diverse ages, educational backgrounds, monthly family incomes, occupations, marital statuses, as well as parity and pregnancy intention. The CHQ score exhibited statistically significant variations among pregnant females of varying monthly family incomes and living sites, as presented in Table 1.

### Correlation analysis of pregnancy stress, mindfulness, peace pf mind, and mental distress of the 713 pregnant women

Results from Pearson’s correlation analysis indicated significant positive correlations at a *p*s < 0.001 level, as presented in Table 2. Specifically, pregnancy stress exhibited a significant positive correlation with mental distress, while displaying significant negative correlations with mindfulness and peace of mind. Furthermore, mindfulness and peace of mind were found to be significantly negatively correlated with mental distress among the pregnant women (Table 2).

**Table 2:** Correlation analysis of study variables of pregnant women (*N* = 713).

Variable	Pregnancy stress	Mindfulness	Peace of mind	Mental distress
Pregnancy stress	1	*--*	--	--
Mindfulness	−0.395***	1	--	--
Peace of mind	−0.403***	0.462***	1	
Mental distress	0.422***	−0.337***	−0.421***	1

***p < 0.001.

### The chain mediating effect of mindfulness and peace of mind in the relationship between pregnancy stress and the mental health of pregnant women

**Figure 2:**
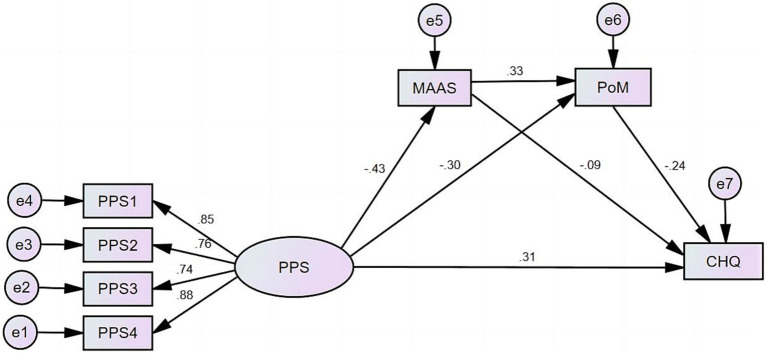
Analysis of chain mediating effect of mindfulness and peace of mind in the relationship between pregnancy stress and mental health of the 713 pregnant women evaluated in this study. PPS, pregnancy pressure scale; PPS1-PPS4, four dimensions (i.e., parent role, mother and child health and safety, body shape and physical activity change, and others) of the PPS; MAAS, Mindful Attention Awareness Scale; SES, self-efficacy scale; PoM, peace of mind; CHQ, Chinese health questionnaire. The same below.

**Figure 3:**
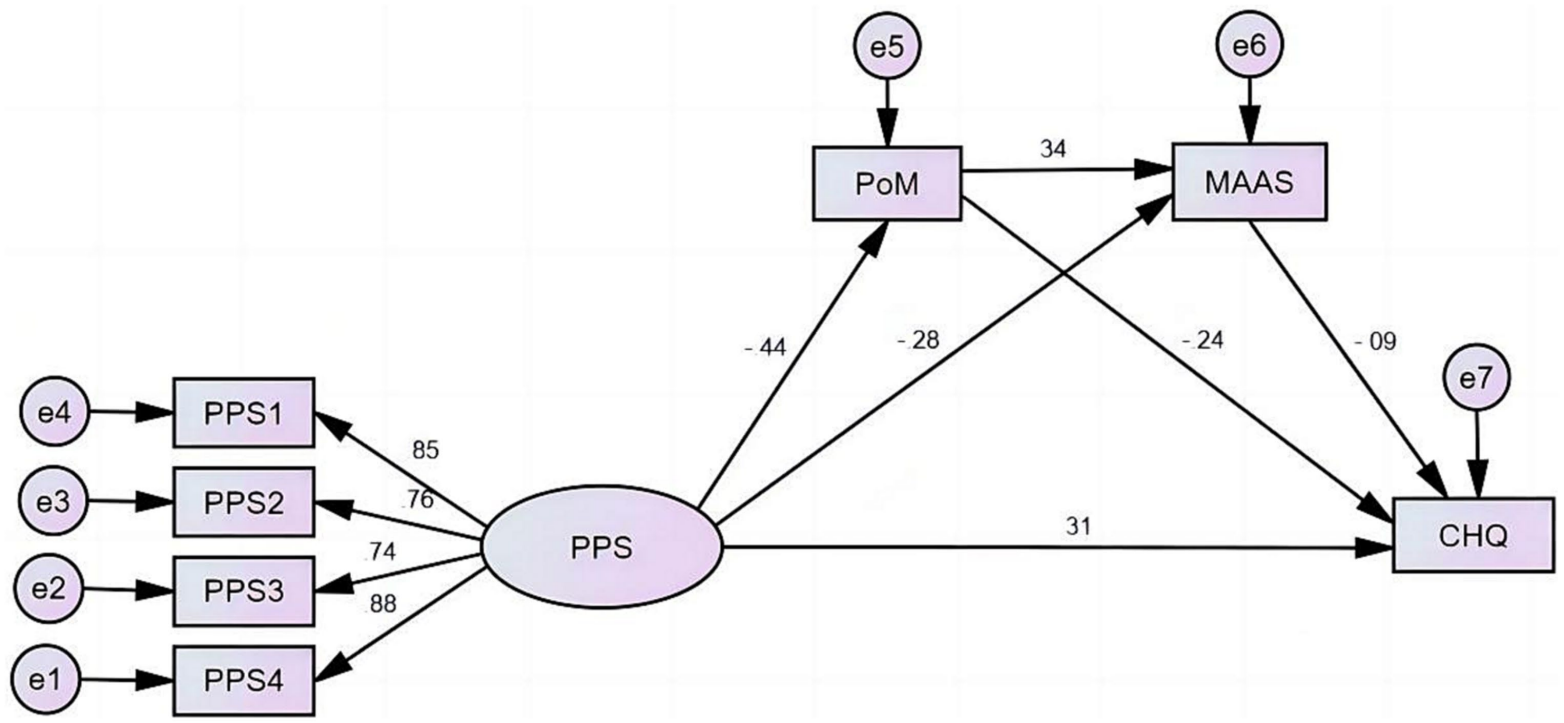
Analysis of chain mediating effects of peace of mind and mindfulness in the relationship between pregnancy stress and mental health of the 713 pregnant women evaluated in this study.

Two SEMs were used to examine the chain mediating impact of mindfulness and peace of mind on the associations between pregnancy stress and mental health among pregnant women. Model 1 (Figure 2) and Model 2 (Figure 3) assessed the chain mediating effects of mindfulness → peace of mind and peace of mind → mindfulness, respectively, in the relationship between pregnancy stress and the mental health of pregnant women, respectively.^3^

Model 1 demonstrated a satisfactory level of fit and achieved statistical significance (*p* < 0.001). The fit indices for this model were as follows: CMIN/DF = 7.316, SRMR = 0.080, RMSEA = 0.094, GFI = 0.941, CFI = 0.912. The results showed that pregnancy pressure had a positive impact on the mental distress experienced by pregnant women (*β* = 0.307, *p* < 0.001). Additionally, pregnancy pressure had a negative influence on both mindfulness (*β* = −0.298, *p* < 0.001) and peace of mind (*β* = −0.430, *p* < 0.001). Conversely, mindfulness (*β* = −0.092, *p* = 0.016) and peace of mind (*β* = −0.238, *p* < 0.000) were found to have negative effects on mental distress of the pregnant women. Model 1 revealed the presence of two simple mediating paths and one chain mediating path. The two mediating effects of PPS → MAAS → CHQ, PPS → PoM → CHQ and the chain effect of PPS → PoM → MAAS → CHQ accounted for 8.63, 15.71 and 7.74% of the total effect, respectively. The chain mediating effect of mindfulness and peace of mind in the relationship between pregnancy stress and mental health of the 713 pregnant women was not significant in the analysis (Table 3).

**Table 3:** Bootstrap analysis of the chain mediating effect of the mindfulness and peace of mind in relationship between pregnancy stress and mental health of the pregnant women.

Path	Effect	SE	95% CI	*p*	Effect value (%)
a1: PPS → MAAS	−0.430	0.040	−0.494 ~ −0.356	0.000	--
a2: MAAS → CHQ	−0.092	0.041	−0.179 ~ −0.012	0.016	--
b1: PPS → PoM	−0.298	0.035	−0.373 ~ −0.216	0.000	--
b2: PoM → CHQ	−0.238	0.042	−0.316 ~ −0.161	0.024	--
c: CHQ → PPS	0.307	0.049	0.208 ~ 0.398	0.000	--
d: MAAS → PoM	0.333	0.037	0.257 ~ 0.403	0.000	--
a1*a2: PPS → MAAS → CHQ	0.039	0.016	0.006 ~ 0.078	0.022	8.63
b1*b2: PPS → PoM → CHQ	0.071	0.018	0.044 ~ 0.106	0.000	15.71
a1*d*b2: PPS → MAAS → PoM → CHQ	0.035	0.008	0.022 ~ 0.051	0.000	7.74
PPS → CHQ (indirect effect)	0.145	0.021	0.072 ~ 0.154	0.000	32.08
PPS → CHQ (direct effect)	0.307	0.049	0.208 ~ 0.398	0.000	67.92
PPS → CHQ (total effect)	0.452	0.041	0.31 ~ 0.493	0.001	100

1. The standardized path coefficients were calculated using the user-defined estimated function in AMOS 23.0, as depicted in Appendix 1. The same procedure was followed subsequently. 2. PPS, pregnancy pressure scale; PPS1-PPS4, four dimensions (i.e., parent role, mother and child health and safety, body shape and physical activity change, and others) of the PPS; MAAS, Mindful Attention Awareness Scale; SES, self-efficacy scale; PoM, peace of mind; CHQ, Chinese health questionnaire. The same below.

### The chain mediating effect of peace of mind and mindfulness in the relationship between pregnancy stress and the mental health of pregnant women

Model 2 demonstrated a satisfactory level of fit and achieved statistical significance (*p* < 0.001). The fit indices for this model were as follows: CMIN/DF = 7.316, SRMR = 0.080, RMSEA = 0.094, GFI = 0.941, CFI = 0.912. The results showed that pregnancy pressure had a positive impact on the mental distress experienced by pregnant women (*β* = 0.307, *p* < 0.001). Additionally, pregnancy pressure had a negative influence on both peace of mind (*β* = −0.442, *p* < 0.001) and mindfulness(*β* = −0.281, *p* < 0.001). Conversely, peace of mind (*β* = −0.238, *p* < 0.001) and mindfulness (*β* = −0.092, *p* = 0.016) were found to have negative effects on the mental distress of pregnant women. Model 1 revealed the presence of two simple mediating paths and one chain mediating path. The two mediating effects of PPS → PoM → CHQ and PPS → MAAS → CHQ, and the chain effect of PPS → PoM → MAAS → CHQ accounted for 23.28, 5.77 and 3.10% of the total effect, respectively (Table 4).

**Table 4:** Bootstrap analysis of the chain mediating effect of the peace of mind and mindfulness in relationship between pregnancy stress and mental health of the pregnant women.

Path	Effect	SE	95% CI	*p*	Effect value (%)
a1: PPS → PoM	−0.442	0.036	−0.509 ~ −0.368	0.000	--
a2: PoM → CHQ	−0.238	0.041	−0.315 ~ −0.156	0.000	--
b1: PPS → MAAS	−0.281	0.039	−0.355 ~ −0.200	0.000	--
b2: MAAS → CHQ	−0.092	0.042	−0.179 ~ −0.012	0.016	--
c: CHQ → PPS	0.307	0.049	0.208 ~ 0.398	0.000	--
d: PoM → MAAS	0.337	0.037	0.263 ~ 0.408	0.000	--
a1*a2: PPS → PoM → CHQ	0.105	0.020	0.068 ~ 0.148	0.000	23.28
b1*b2: PPS → MAAS → CHQ	0.026	0.012	0.004 ~ 0.053	0.021	5.77
a1*d*b2: PPS → PoM → MAAS → CHQ	0.014	0.007	0.002 ~ 0.028	0.020	3.10
PPS → CHQ (indirect effect)	0.145	0.023	0.102 ~ 0.192	0.000	32.15
PPS → CHQ (direct effect)	0.306	0.049	0.213 ~ 0.395	0.000	67.85
PPS → CHQ (total effect)	0.451	0.041	0.368 ~ 0.526	0.000	100

## Discussion

Consistent with previous studies (Coussons-Read, 2013; Parftt and Ayers, 2014; Puertas-Gonzalez et al., 2022; Tuxunjiang et al., 2022, 2023), the current research also finds a notable correlation between stress during pregnancy and psychological distress in pregnancy women, indicating that pregnancy stress plays a role in aggravating their mental health. The present investigation, from the standpoint of positive psychology, further examined how mindfulness and peace of mind influenced the connection between stress during pregnancy and the mental well-being of expectant mothers. Mindfulness and peace of mind act as mediators for pregnancy stress and mental health, as demonstrated in Model 1 and Model 2. Moreover, the practice of being present and having a calm state of mind have a reciprocal influence on the connection between stress during pregnancy and the mental well-being of pregnancy women. This research is the initial investigation into the impact of peace of mind on the correlation between stress during pregnancy and the psychological well-being of pregnancy women.

### Theoretical implications

First, the initial finding of this study demonstrates the significant mediating effect of mindfulness, suggesting its potential to mitigate the negative impact of stress during pregnancy on mental health outcomes. Mindfulness, as a positive psychological quality, has garnered increasing attention from both the general public and researchers in recent years. Numerous studies have also explored and confirmed the beneficial impact of mindfulness in promoting the well-being of pregnant women (Braeken et al., 2017; Zhang et al., 2019; Tuxunjiang et al., 2022, 2023; De Waal et al., 2023). This current study successfully replicates these findings within a Chinese sample.

Second, the present research finds a significant indirect impact of stress during pregnancy on mental well-being through the intermediary aspect of peace of mind. This aligns with the theoretical framework, which draws upon the broaden-and-build theory of positive emotions (Fredrickson and Branigan, 2005) and the conservation of resource (COR) theory (Halbesleben et al., 2014). These findings reinforce earlier investigations that emphasized the distinct benefits of peace of mind in cultivating and preserving personal resources (Yu et al., 2019; Zhou et al., 2021), as well as its efficacy in managing intense anger or coping with cancer (Gittzus et al., 2020; Saputra et al., 2021). Going beyond replication, this study expands upon existing research by presenting empirical evidence that peace of mind may serve as a positive psychological quality and a significant mechanism for mitigating the adverse impacts of pregnancy-related stress, thereby enhancing the mental well-being of expectant mothers.

A noteworthy finding of the present study is that the influence of peace of mind in explaining the variances of the models is more dominant than that of mindfulness in both Model 1 (15.71% versus 8.63%) and Model 2 (23.28% versus 5.77%). This suggests that, in terms of the association between pregnancy stress and the mental health of pregnant women, peace of mind appears to be a more effective strategy than mindfulness in mitigating the negative effects of pregnancy stress. Further research is warranted to investigate the stability of these findings. Despite the widespread attention and research on mindfulness, it is important to consider the potential benefits of peace of mind in this context.

Third, this study is one of the initial investigations to examine the interaction between mindfulness and peace of mind, and their impact on the correlation between stress during pregnancy and the mental well-being of the pregnant women. The results demonstrate that mindfulness and peace of mind play a reciprocal role in mediation. Crucially, the findings suggest that the mental well-being of the pregnant women is not solely mitigated by either mindfulness or peace of mind in isolation, but rather necessitates the synergistic interaction of these two crucial positive psychological qualities.

The observed correlation between mindfulness and peace of mind aligns with the underlying mechanisms of mindfulness, which involve the deliberate cultivation of nonjudgmental attention, fostering self-regulation, and enhancing mental well-being (Shapiro et al., 2006). Scholars argue that peace of mind may serve as a psychological resource that facilitates adaptive functioning, potentially elucidating the direct impact of peace of mind on mindfulness (Lee et al., 2013; Soysa et al., 2021; Niemiec, 2022). Additional research is necessary to elucidate the specific ways in which peace of mind contributes to mindfulness and mental health of pregnant women.

The main theoretical importance of this research is in confirming the reciprocal link between mindfulness and peace of mind, which acts as a mediator in the connection between stress during pregnancy and the mental well-being of the pregnant women. From the positive psychology standpoint, this research illuminates the hidden safeguarding mechanism of stress during pregnancy on mental well-being. The results highlight the crucial importance of mindfulness and peace of mind, as beneficial positive psychological qualities, in reducing stress and enhancing emotional wellness throughout pregnancy.

### Practical implications

This study is expected to make a significant contribution to the understanding of the impact of mindfulness and peace of mind on reducing pregnancy stress and promoting mental health among pregnant women. Interventions focused on cultivating mindfulness during pregnancy have the potential to be advantageous in mitigating pregnancy-related stress and improving the psychological well-being of pregnant women, as evidenced by previous studies (Zhang et al., 2019; Tuxunjiang et al., 2022, 2023; De Waal et al., 2023). Furthermore, based on the findings of the current study, it appears that peace of mind emerges as a more efficacious approach than mindfulness in alleviating the detrimental impacts of stress during pregnancy. This aligns with the assertions made by Soysa et al. (2021), highlighting that peace of mind provides a frame of reference that enables individuals to cultivate appreciation toward life’s offerings, consequently empowering them to confront challenges with serenity and composure. Consequently, pregnant women may potentially benefit from targeted interventions that are specifically designed to enhance tranquility and inner harmony, thereby promoting their overall well-being (Soysa et al., 2021; Niemiec, 2022; Xi et al., 2023). Considering the reciprocal link between mindfulness and peace of mind in the context of pregnancy stress and mental health, it is recommended that further mindfulness-based interventions (MBIs) implemented for pregnant women should assess variables such as mindfulness, peace of mind, emotional stability, and emotional regulation abilities. This approach aims to elucidate the underlying mechanisms through which mindfulness and peace of mind exert influence on the overall mental health of pregnant individuals (Xi et al., 2023).

In addition to interventions aimed at enhancing mindfulness and peace of mind among pregnant women, the inclusion of psychological education is crucial. It is recommended that interventions designed for pregnant women incorporate the framework of positive psychology, along with theories such as the broaden-and-build theory of positive emotions (Fredrickson and Branigan, 2005) and the conservation of resource (COR) theory (Halbesleben et al., 2014). Within the Chinese cultural context, there is a belief that well-being is characterized by reserved low-arousal affects, as opposed to emotionally charged high-arousal affects. The cultivation of peace of mind is believed to offer distinct benefits in the development and preservation of personal resources (Yu et al., 2019; Zhou et al., 2021). These theories and beliefs should be integrated to help pregnant women comprehend the underlying mechanisms that motivate them to maintain mindfulness and tranquility. Numerous studies have demonstrated the effectiveness of psychological education in alleviating pregnancy-related stress (Yazdanimehr et al., 2016; Yang et al., 2019; Aguilera-Martín et al., 2021).

### Limitations and future research

There were several limitations of the current study. First, the research employed a cross-sectional approach, assessing all factors concurrently, thereby preventing the determination of temporal connections and the inference of causation. In order to overcome this constraint, forthcoming studies should utilize experimental and longitudinal methodologies to explore the possible causal function of peace of mind in the connection between mindfulness and its impacts, along with the influence of stress during pregnancy on mental health of the pregnant women. Second, additional investigations are necessary to explore the mechanisms and contextual constraints related to the impact of peace of mind on the psychological welfare of the pregnant women. For example, the mental well-being of expectant mothers may be influenced by factors such as emotional stability and the ability to control their emotions, which are important mechanisms underlying the impact of peace of mind on their overall mental health (Xi et al., 2023). Third, it is crucial to acknowledge that this research exclusively concentrated on Chinese participants, thereby overlooking the investigation of possible differences in peace of mind among individuals from diverse nations or circumstances. According to the assertion made by Lee et al. (2013), the importance given to peace of mind may vary among different cultures, especially in societies that prioritize collective values. Consequently, additional research examining cultural diversity is imperative. Finally, although the current research effectively validated the reciprocal role of mindfulness and peace of mind in relationship between pregnancy stress and mental health of the pregnant women, additional inquiries are required to clarify the complex connection between the two important positive psychological qualities.

## Data availability statement

The original contributions presented in the study are included in the article/Supplementary material, further inquiries can be directed to the corresponding author/s.

## Ethics statement

The studies involving humans were approved by Clinical Research and Experimental Animal Ethics Committee, the First Affiliated Hospital of Sun Yat-Sen University. The studies were conducted in accordance with the local legislation and institutional requirements. The participants provided their written informed consent to participate in this study.

## Author contributions

SS: Data curation, Formal analysis, Methodology, Writing – original draft, Writing – review & editing. CL: Data curation, Investigation, Writing – original draft. QW: Formal analysis, Methodology, Writing – review & editing. XZ: Conceptualization, Supervision, Writing – review & editing.

## Footnotes

^1^https://www.wjx.cn/

^2^The 12 items of the CHQ are presented in Appendix 2.

^3^In both Model 1 and Model 2, the demographic variables of age, educational backgrounds, and monthly family income were controlled, and the pathways of these variables were not statistically significant.
