# Supplementary material for: The relationship between pregnancy stress and mental health of the pregnant women: the bidirectional chain mediation roles of mindfulness and peace of mind
Source: Front Psychol. 2024 Jan 8;14:1295242. doi: 10.3389/fpsyg.2023.1295242 (PMC10800386; doi:10.3389/fpsyg.2023.1295242)

Appendix 1

**Model 1**

Code:

stda1=e.StandardizedDirectEffect(MAAS,PPS)

stda2=e.StandardizedDirectEffect(CHQ,MAAS)

stdb1=e.StandardizedDirectEffect(PoM,PPS)

stdb2=e.StandardizedDirectEffect(CHQ,PoM)

stdc=e.StandardizedDirectEffect(CHQ,PPS)

stdd=e.StandardizedDirectEffect(PoM,MAAS)

stdind1=stda1*stda2

stdind2=stdb1*stdb2

stdind3=stda1*stdd*stdb2

stdtotind=stdind1+stdind2+stdind3

stdtotal=stdc+stdtotind

Parameter name:


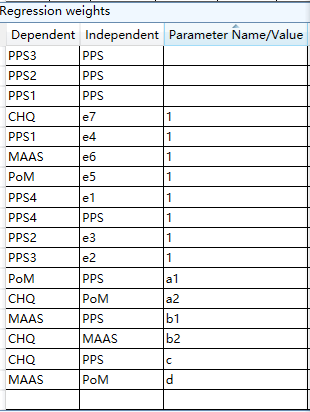


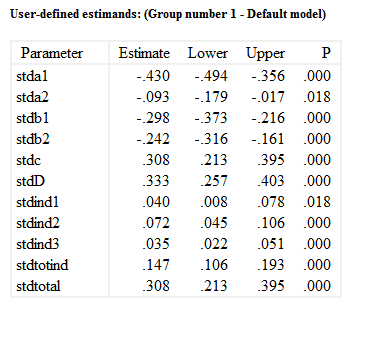


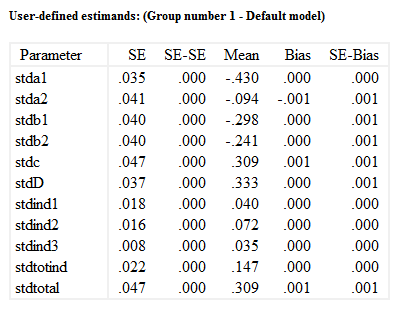


**Model 2**

Code:

stda1=e.StandardizedDirectEffect(PoM,PPS)

stda2=e.StandardizedDirectEffect(CHQ,PoM)

stdb1=e.StandardizedDirectEffect(MAAS,PPS)

stdb2=e.StandardizedDirectEffect(CHQ,MAAS)

stdc=e.StandardizedDirectEffect(CHQ,PPS)

stdd=e.StandardizedDirectEffect(MAAS,PoM)

stdind1=stda1*stda2

stdind2=stdb1*stdb2

stdind3=stda1*stdd*stdb2

stdtotind=stdind1+stdind2+stdind3

stdtotal=stdc+stdtotind

Parameter name:


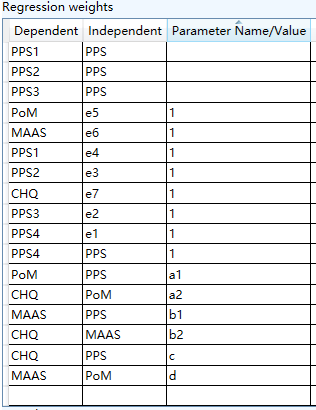


Results:


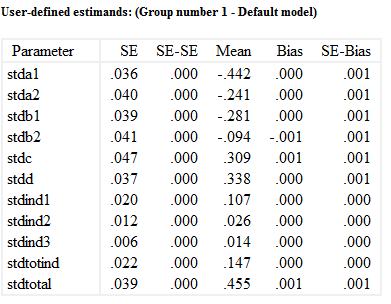


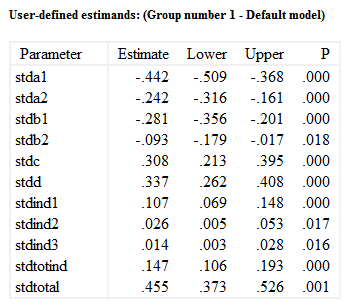

Supplement: Supplementary file 1 [file Table_1.DOCX]
