# Supplementary material for: The relationship between pregnancy stress and mental health of the pregnant women: the bidirectional chain mediation roles of mindfulness and peace of mind
Source: Front Psychol. 2024 Jan 8;14:1295242. doi: 10.3389/fpsyg.2023.1295242 (PMC10800386; doi:10.3389/fpsyg.2023.1295242)
Supplement: Supplementary file 2 [file Table_2.DOC]

Items of CHQ

CHQ 01 been suffering from headache or pressure in your head?

CHQ 02 had palpitations and worried that you might have a heart trouble?

CHQ 03 had discomfort or a feeling of pressure in your chest?

CHQ 04 been suffering from shaking or numbness of your limbs?

CHQ 05 lost much sleep over worry?

CHQ 06 been taking things hard?

CHQ 07 been getting along well with your family and close relatives?

CHQ 08 been losing confidence in yourself?

CHQ 09 been feeling nervous and highstrung?

CHQ 10 been feeling hopeful about your future?

CHQ 11 been worrying about your family or close relatives?

CHQ 12 been feeling that life is entirely hopeless?

华人健康问卷

以下题目关于你的健康状况，请选择最近一个月以来最符合你情况的选项。

1表示「一点也不」，4 分表示「比平时更觉得」

1. 觉得头痛或头部有压迫感

2. 心悸或担心心脏有问题

3. 胸部不适或有压迫感

4. 颤抖或四肢麻木

5. 因担忧而失眠

6. 觉得想不开

7. 与家人和朋友相处得来

8. 对自己失去信心

9. 觉得神经紧绷、紧张不安

10. 对未来充满希望

11. 担心自己的家庭或亲朋好友会出什么事

12. 认为生活完全没有希望
